# Supplementary material for: DNA polymerase ζ is a robust reverse transcriptase
Source: bioRxiv. 2024 Sep 28:2024.09.27.615452. Preprint. [Version 1] doi: 10.1101/2024.09.27.615452 (PMC11463433; doi:10.1101/2024.09.27.615452)
Supplement: 1 [file NIHPP2024.09.27.615452V1-supplement-1.pdf]

bioRxiv preprint doi: https://doi.org/10.1101/2024.09.27.615452; this version posted September 28, 2024. The copyright holder for this preprint (which was not certified by peer review) is the author/funder, who has granted bioRxiv a license to display the preprint in perpetuity. It is made available under aCC-BY 4.0 International license.

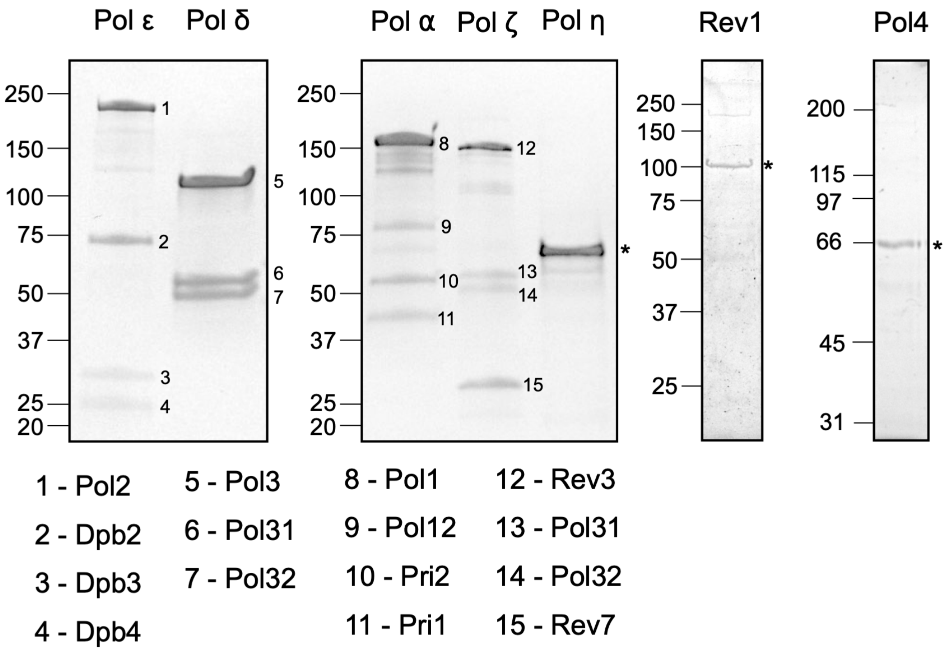

Figure S1: SDS-PAGE gels showing each of the purified yeast polymerases: Bands for individual proteins are indicated by either a number, for multi-subunit enzymes, or a * for single subunit polymerases.

bioRxiv preprint doi: https://doi.org/10.1101/2024.09.27.615452; this version posted September 28, 2024. The copyright holder for this preprint (which was not certified by peer review) is the author/funder, who has granted bioRxiv a license to display the preprint in perpetuity. It is made available under aCC-BY 4.0 International license.

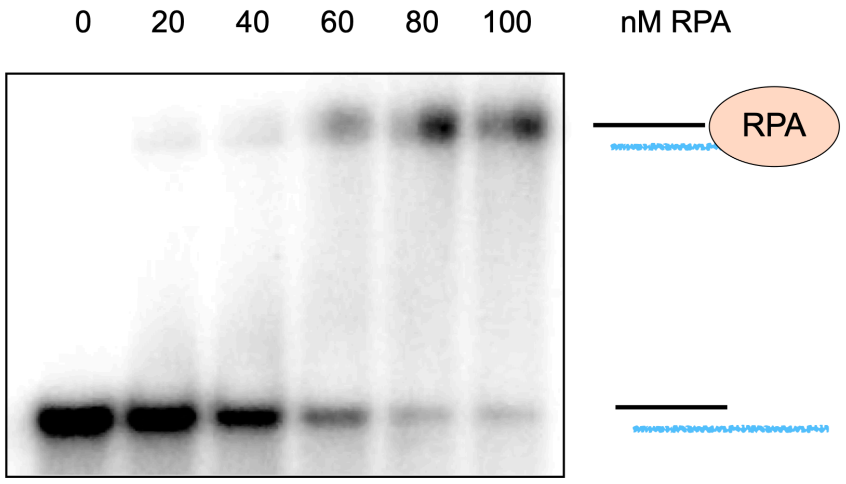

Figure S2. RPA binds the DNA primed RNA substrate: A gel shift assay after incubation of RPA with the DNA primed RNA substrate for 5 minutes at 30°C.

bioRxiv preprint doi: https://doi.org/10.1101/2024.09.27.615452; this version posted September 28, 2024. The copyright holder for this preprint (which was not certified by peer review) is the author/funder, who has granted bioRxiv a license to display the preprint in perpetuity. It is made available under aCC-BY 4.0 International license.

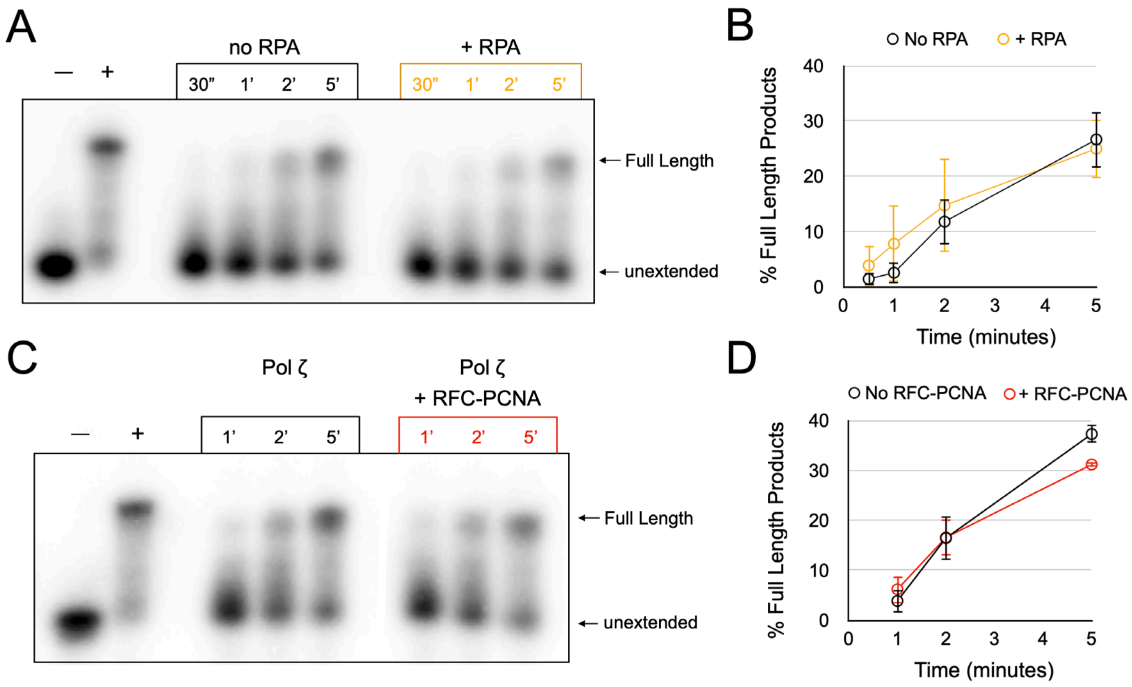

Figure S3. RPA and RFC/PCNA have no notable impact on Pol ζ RT activity: (A) shows primer extension RT assays comparing the presence and absence of RPA, and **(B)** is a quantitation of triplicate assays. Error bars represent +/- standard error of the mean. **(C)** shows primer extension RT assays comparing the presence and absence of RFC/PCNA. **(D)** is a quantitation of triplicate assays. Error bars represent +/- standard error of the mean.

bioRxiv preprint doi: https://doi.org/10.1101/2024.09.27.615452; this version posted September 28, 2024. The copyright holder for this preprint (which was not certified by peer review) is the author/funder, who has granted bioRxiv a license to display the preprint in perpetuity. It is made available under aCC-BY 4.0 International license.

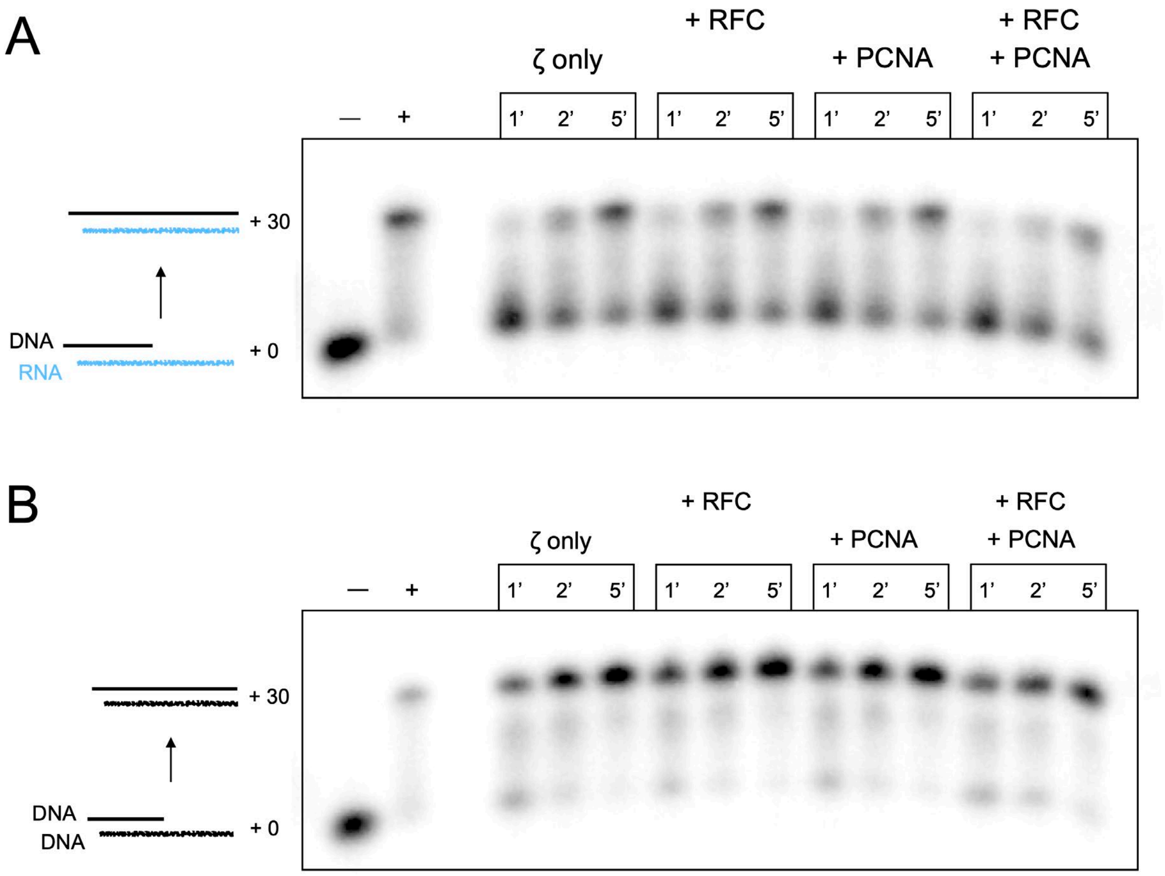

Figure S4: RFC +/- PCNA does not stimulate Pol ζ on either DNA or RNA templates: Extension assays with the noted additions of RFC and/ or PCNA using either RNA (A) or DNA (B) template strands.
